# Combined Oat β-Glucan and Soy Protein Isolate Reprogram Gut Microbiota and Improve Metabolic Dysfunction in Diet-Induced Obesity

**DOI:** 10.3390/nu18101571

**Published:** 2026-05-15

**Authors:** Zongzhen Guo, Yuge Sun, Yiyun Zhang, Kefan Wei, Jiaqian Cao, Qun Shen, Yong Xue

**Affiliations:** 1 National Engineering and Technology Research Center for Fruits and Vegetables, College of Food Science and Nutritional Engineering, China Agricultural University, Beijing 100083, China; 2 National Center of Technology Innovation (Deep Processing of Highland Barley) in Food Industry, China Agricultural University, No. 17 Qinghua East Road, Haidian District, Beijing 100083, China

**Keywords:** gut–liver axis, metabolic reprogramming, gut microbiota, β-glucan, soy protein, insulin resistance, hepatic steatosis, *Akkermansia*

## Abstract

Background/Objectives: Although plant-derived dietary fiber and protein are favorable factors for improving host metabolic disorders, it remains unclear whether these two macronutrients exhibit synergistic health benefits. Methods: To address this gap, utilizing oat dietary fiber (GLU) and soybean protein (SBP) as representative bioactive models, we investigated the effects of 5% GLU, 20% SBP, and their combined supplementation on high-fat diet (HFD)-induced metabolic dysregulation in C57BL/6J mice. Results: Our results demonstrated that the combined GLU + SBP intervention provided comprehensive protection against HFD-induced obesity, significantly attenuating body weight gain (12.29 ± 2.02 g vs. 21.90 ± 2.86 g, *p* < 0.05) and adiposity (3.34 ± 1.19% vs. 10.77 ± 1.16%, *p* < 0.05) compared with HFD mice, without altering caloric intake. Crucially, the compound formulation exhibited synergistic superiority over individual components, as evidenced by greater reductions in serum aspartate aminotransferase (AST) activity (113.13 ± 28.50 U/L vs. 158.00 ± 30.25 U/L, *p* < 0.05) and improved glucose tolerance, with lower OGTT AUC values (999.09 ± 95.83 vs. 1434.66 ± 80.56 mmol/L·min, *p* < 0.05). Mechanistically, 16S rRNA sequencing revealed a distinct remodeling of the gut microbial community, highlighted by a substantial enrichment of *Akkermansia*. Functional prediction analysis specifically linked this microbial shift to the modulation of *Akkermansia*-associated metabolic pathways, which subsequently facilitated the activation of host metabolic networks to combat lipid deposition and systemic metabolic stress. Conclusions: Collectively, the GLU + SBP combination offers synergistic metabolic benefits driven by a distinct gut microbiota signature, supporting a feasible “soluble fiber + plant protein” strategy for developing functional foods targeting metabolic health.

## 1. Introduction

Obesity has emerged as a major global public health challenge and is closely associated with a range of metabolic complications, including dyslipidemia, insulin resistance and metabolic dysfunction-associated fatty liver disease (MAFLD) [1,2,3,4]. Although pharmacological and surgical interventions offer clinical benefits for specific populations with obesity, scalable and sustainable population-level prevention largely depends on dietary strategies that enhance metabolic health. In this regard, plant-derived foods emerge as highly promising candidates, as they provide a synergistic matrix of dietary fibers, proteins, unsaturated lipids, and phytochemicals [5,6]. These components may act in a complementary manner to modulate host metabolic pathways and reshape the composition and function of gut microbiota, thereby influencing energy homeostasis, glucose metabolism, lipid transport and intestinal barrier integrity [7,8]. Consistent with these regulatory effects, higher intake of whole grain has been shown to improve long-term weight management and reduce cardiometabolic risk profiles, as supported by rigorous prospective cohort studies and systematic evaluations of dietary patterns [9,10,11]. Despite these consistent epidemiological evidences, the mechanistic basis underlying the synergistic and complementary interactions among dietary components remains insufficiently understood, highlighting the need for well-controlled experimental studies.

Plant-derived β-glucans and proteins are two major classes of dietary macromolecules whose metabolic benefits are supported by both animal models and human studies [12,13], making them strong candidates for exploring the mechanisms underlying dietary improvement in obesity-related interventions. Beta-glucan, a dietary fiber enriched in oats and barley [14], has demonstrated consistent lipid-lowering effects in animal studies and meta-analyses of human intervention trials. Beyond circulating lipids, diet-induced fatty liver disease models further suggest that oat β-glucan can attenuate hepatic lipid accumulation and liver injury, often in conjunction with remodeling of the gut microbiota, implicating a gut–liver axis mechanism relevant to MAFLD [15]. Mechanistic studies in high-fat diet (HFD)-fed mice also indicate that β-glucan may ameliorate insulin resistance and intestinal barrier dysfunction, providing a plausible link to improved glucose homeostasis [16]. Similarly, soy protein has been associated with more favorable atherogenic lipid profiles in meta-analyses of human studies and has showed protective effects against hepatic steatosis and inflammation in obesity-related mouse models [17,18], frequently alongside shifts in gut microbial composition. In MAFLD-relevant clinical settings, soy-based interventions have also been reported to improve liver enzymes and related metabolic markers [19]. However, as the majority of studies have investigated β-glucan or soy protein individually under HFD conditions, the extent to which their combined administration produces additive or synergistic effects remains to be clearly established.

The gut microbiota has emerged as a critical mediator of diet–host metabolic crosstalk [20,21,22]. HFD exposure induces reproducible shifts in microbial community structure and function, with consequences for metabolic endotoxemia, low-grade inflammation, insulin resistance, and hepatic lipid handling [23]. Within this framework, enrichment of *Verrucomicrobiota*—particularly the genus *Akkermansia*—has repeatedly been associated with improved metabolic phenotypes. In mouse models, *Akkermansia muciniphila* administration mitigates HFD-induced hyperglycemia, lipid accumulation, and insulin resistance, while early human intervention studies have demonstrated an acceptable safety profile alongside evidence of weight loss and reduced blood glucose levels, these findings support its relevance as both a biomarker and a candidate effector taxon [24]. Conversely, sulfate-reducing bacterial taxa, including *Desulfovibrionaceae*, are prominently implicated in HFD-associated inflammatory and hepatic dysfunction. Although the functional outcomes of these taxa exhibit contextual variability, recent investigations demonstrate that *Desulfovibrio piger* exacerbates hepatic steatosis and fibrosis in HFD-fed mice, with parallel observations of increased abundance in pediatric MAFLD cohorts-findings that underscore its potential significance as a HFD-responsive microbial marker [25]. These observations support a mechanistic hypothesis that co-administration of β-glucan and soy protein may reshape the gut–liver axis by enriching beneficial soy protein-associated taxa, suppressing HFD-associated microbial signatures, and altering microbial metabolic profiles. Direct experimental validation is still required to establish causal relationships.

In this study, we selected oats and soybeans—representative staple crops [26,27]—as readily available sources of soluble dietary fiber and plant protein. Our objective was to determine whether combined supplementation with oat β-glucan and soy protein isolate produces synergistic effects on HFD-induced metabolic dysfunction in C57BL/6J mice. An isocaloric HFD-matched control diet was administered to assess body weight gain, fat accumulation, serum lipid profiles, hepatic steatosis, and glucose metabolism. Gut microbial community restructuring was analyzed via 16S rRNA amplicon sequencing to identify taxonomic shifts and key microbial signatures in response to individual versus combined interventions. The correlation of gut microbiota at genus level with host physiological markers was analysis, and microbial functional profiles were predicted using PICRUSt2 and, with the caveat that 16S-derived functional inferences serve exploratory purposes requiring experimental validation [28]. Collectively, this comprehensive approach establishes a framework for the rational design of “dietary fiber-plant protein” combinations aimed at mitigating obesity-related metabolic disorders through mechanistic insights into host-microbe interactions.

## 2. Materials and Methods

### 2.1. Materials and Reagents

Oat β-glucan (purity ≥ 80%) was purchased from Xi’an Lavia Biotechnology Co., Ltd. (Xi’an, China). Soybean protein isolate (protein content ≥ 87%) was obtained from Jilin Yirun Protein Co., Ltd. (Songyuan, China). The fundamental nutrient compositions of these two functional ingredients were analyzed according to Chinese National Standards and are presented in Table S1. Commercial assay kits for total cholesterol (TC), triglycerides (TG), high-density lipoprotein cholesterol (HDL-C), low-density lipoprotein cholesterol (LDL-C), aspartate aminotransferase (AST), and alanine aminotransferase (ALT) were purchased from Nanjing Jiancheng Bioengineering Institute (Nanjing, China). All the other chemicals and solvents used were of analytical grade.

### 2.2. Diet Preparation

Five experimental diets were formulated based on the diets D12450J (10% kcal from fat) and D12492 (60% kcal from fat): (1) Normal control diet (NCD); (2) High-fat diet (HFD); (3) HFD supplemented with oat β-glucan (GLU); (4) HFD supplemented with soybean protein isolate (SBP); and (5) HFD supplemented with both oat β-glucan and soybean protein isolate (GLU + SBP). The interventional diets were supplemented with 5% β-glucan [29], 20% soybean protein [30], or a combination of both. The 5% dosage of β-glucan was selected based on our preliminary observations and previous HFD-fed mouse studies to produce measurable improvements in HFD-induced metabolic disturbances. While the supplementation of 30% (*w*/*w*) whole oats in a diet provides only ~1.79% pure β-glucan (based on an extraction yield of 5.96%), this low concentration fails to reach the effective threshold required to mitigate HFD-induced metabolic disorders. Consequently, a standardized dose of 5% β-glucan was utilized to achieve an optimal and observable therapeutic effect.

To ensure comparability among the HFD-based diets, all HFD groups (HFD, GLU, SBP, and GLU + SBP) were formulated to be isocaloric and to have matched macronutrient-derived energy ratios (20% protein, 20% carbohydrate, and 60% fat), whereas the NCD group provided 20% protein, 70% carbohydrate, and 10% fat. In the GLU and GLU + SBP diets, the added β-glucan mainly replaced a portion of cellulose and maltodextrin. In the SBP and GLU + SBP diets, SPI substituted a corresponding amount of casein to maintain consistent total protein levels. The detailed ingredient composition and energy ratios of the experimental diets are shown in Table 1. All diets were processed by a professional animal feed manufacturer (China Agricultural University, Beijing, China) and stored at 4 °C until use.

### 2.3. Animal Experiments and Ethics

Male C57BL/6J mice (4 weeks old, SPF grade) were obtained from Beijing Vital River Laboratory Animal Technology Co., Ltd. The animal study protocol was approved by the Animal Care and Use Committee of China Agricultural University (Approval No. AW2202202-4-1). Mice were housed in a specific pathogen-free facility under controlled conditions (22 °C ± 2 °C, 55% ± 5% humidity, 12 h light/dark cycle) with ad libitum access to food and water. After 1 week of acclimatization, 40 mice were randomly divided into five groups (*n* = 8 per group) based on body weight to ensure no initial significant differences. The mice were fed their respective experimental diets for 12 weeks.

Throughout the intervention period, body weight and food intake were recorded twice weekly throughout the intervention period. Body weight gain was calculated as the difference between the final and baseline body weights (Week 12 − Week 0). Food intake was expressed as average daily food intake (g/day) per mouse, calculated as the difference between the provided and remaining feed over a defined period, and normalized by the number of mice and days. Daily energy intake (kcal/day) was calculated by multiplying daily food intake (g/day) by the metabolizable energy density (kcal/g) of each diet provided by the manufacturer (Table 1).

### 2.4. Oral Glucose Tolerance Test

An oral glucose tolerance test (OGTT) was conducted in the 11th week of the intervention. Mice were fasted for 6 h, followed by oral gavage of a glucose solution (2.0 g/kg body weight). Blood samples were collected from the tail vein at 0, 15, 30, 60, 90 and 120 min. Then, blood glucose levels were measured using a glucometer (Bayer, Leverkusen, Germany). The area under the curve (AUC) for glucose was calculated for glucose tolerance evaluation. Fasting blood glucose was measured after a 6 h fast using tail vein blood and a glucometer (Bayer, Leverkusen, Germany). The fasting value corresponded to the 0 min time point of the OGTT [16].

### 2.5. Sample Collection

At the end of the 12-week intervention, mice were fasted for 12 h. Blood samples were collected via retro-orbital sinus puncture, centrifuged at 3000 rpm for 15 min at 4 °C to obtain serum, and then stored at −80 °C for biochemical analysis. Subsequently, mice were sacrificed via cervical dislocation. The liver and white adipose tissues (epididymal, perirenal, and subcutaneous fat) were carefully dissected, weighed, and either snap-frozen in liquid nitrogen for downstream analyses or fixed in 4% paraformaldehyde for histological examination. White adipose tissue depots, including epididymal (Epi-WAT), perirenal (Per-WAT), and retroperitoneal (Retro-WAT) fat masses, were weighed. Total fat mass was calculated as the sum of these depots.

### 2.6. Biochemical Analysis

The serum levels of TC, TG, HDL-C, LDL-C, AST, and ALT were determined using commercial enzymatic kits (Nanjing Jiancheng Bioengineering Institute, Nanjing, China) according to the manufacturer’s instructions [18].

### 2.7. Histological Analysis

Liver tissues were fixed in 4% paraformaldehyde, dehydrated using a graded ethanol series, cleared with xylene, embedded in paraffin, and sectioned at 5-μm thickness. Paraffin sections were stained with hematoxylin and eosin to evaluate general hepatic architecture and steatosis-related morphological changes. Representative H&E images were captured using a biological microscope (Nikon, Tokyo, Japan), with a scale bar of 100 μm (Figure 2E). For lipid accumulation assessment, a portion of liver tissue was processed for frozen sectioning and stained with Oil Red O for the visualization of neutral lipid deposition. Representative Oil Red O images were captured under identical microscope settings, with a 50-μm scale bar (Figure 2F). Oil Red O staining was used for qualitative assessment, and no image-based quantification was performed in the present study [15].

### 2.8. Gut Microbiota Analysis

#### 2.8.1. DNA Extraction and 16S rRNA Sequencing

Fecal samples were collected at Week 11 and immediately stored at −80 °C until analysis. Total genomic DNA was extracted using the E.Z.N.A.^®^ Soil DNA Kit (Omega Bio-Tek, Norcross, GA, USA) according to the manufacturer’s protocol. DNA concentration and purity were evaluated using a NanoDrop 2000 spectrophotometer (Thermo Fisher Scientific, Waltham, MA, USA). The V3–V4 hypervariable regions of the bacterial 16S rRNA gene were amplified using primers 338F (5′-ACTCCTACGGGAGGCAGCAG-3′) and 806R (5′-GGACTACHVGGGTWTCTAAT-3′). Amplicons were purified, quantified, pooled in equimolar concentrations, and sequenced on an Illumina MiSeq PE300 platform (Illumina, San Diego, CA, USA) [31].

#### 2.8.2. Bioinformatics Analysis

Raw sequencing data were quality-filtered and clustered into operational taxonomic units (OTUs) at 97% similarity using UPARSE. Taxonomy was assigned using the RDP classifier against the SILVA reference database. Alpha diversity indices (e.g., Chao1 and Shannon) were calculated using QIIME and visualized using R 4.2.0. Beta diversity was evaluated using the Bray–Curtis dissimilarity matrix at the OTU level. The microbial community structure was visualized using principal coordinate analysis and nonmetric multidimensional scaling. The group differences in overall community composition were tested using permutational multivariate analysis of variance (PERMANOVA) based on Bray–Curtis distances. Linear discriminant analysis effect size (LEfSe) was used to identify differentially abundant taxa, with taxa considered significant if they had linear discriminant analysis (LDA) score > 3.0 and *p* < 0.05. The Kyoto Encyclopedia of Genes and Genomes (KEGG) pathway enrichment analysis was used to analyze functional enrichment clusters. Functional prediction of the gut microbiota was performed using PICRUSt2 [28], and predicted functional profiles/pathways were compared among the groups. Where applicable, effect size estimates with confidence intervals were used to present differences in predicted pathway abundances.

### 2.9. Statistical Analysis

Data are presented as mean ± SEM unless otherwise stated. Statistical analyses were performed using SPSS 26.0 (IBM, Armonk, NY, USA) and GraphPad Prism 9.0 (GraphPad Software, San Diego, CA, USA). Differences among groups were evaluated using one-way analysis of variance (ANOVA) followed by Duncan’s test. OGTT time-course data were analyzed using two-way repeated-measures ANOVA (time × treatment), while OGTT AUC values were compared by one-way ANOVA. A *p*-value < 0.05 was considered statistically significant. Spearman’s correlation analysis was used to evaluate associations between gut microbiota (at the genus level) and metabolic parameters, and correlation heatmaps were generated in R 4.2.0.

## 3. Results

### 3.1. Dietary Combination of Oat β-Glucan and Soybean Protein Alleviates HFD-Induced Obesity and Dyslipidemia

**Figure 1:**
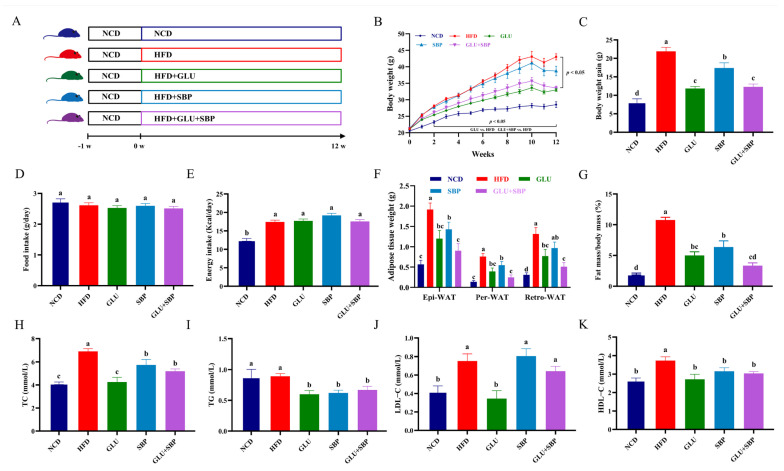
Experimental design and effects of GLU + SBP on phenotypic parameters and lipid profiles. (**A**) Schematic diagram of the experimental flow and timeline. (**B**) Body weight change curves during the 12-week intervention. (**C**) Net body weight gain. (**D**) Average daily food intake (g/day). (**E**) Average daily energy intake (kcal/day). (**F**) Absolute weights of white adipose tissues (WAT). (**G**) Ratio of white adipose tissue to body weight (fat coefficient). (**H**–**K**) Serum lipid profiles: (**H**) TC, (**I**) TG, (**J**) LDL-C, and (**K**) HDL-C. Data are expressed as mean ± SD (*n* = 8). Different letters indicate statistically significant differences between the groups (*p* < 0.05) determined by Duncan’s test. NCD: normal control diet, HFD: high-fat diet, GLU: HFD + β-glucan, SBP: HFD + soybean protein, GLU + SBP: HFD + β-glucan and soybean protein combination.

Figure 1A summarizes the experimental workflow and dietary interventions. HFD feeding induced a clear obese phenotype, as reflected by a sustained increase in body weight over the 12-week period (Figure 1B) and a markedly greater net body weight gain compared with the NCD group (Figure 1C). Dietary supplementation with oat β-glucan (GLU), soybean protein isolate (SBP), or their combination (GLU + SBP) attenuated HFD-induced weight gain. In particular, the GLU + SBP group exhibited significantly lower final body weight and net body weight gain than the HFD group. Final body weight was reduced from 40.30 ± 1.41 g in HFD-fed mice to 35.33 ± 0.99 g in GLU + SBP-treated mice (*p* < 0.05), and net body weight gain was reduced from 21.90 ± 2.86 g to 12.29 ± 2.02 g (*p* < 0.05) (Figure 1B,C). To determine whether these phenotypic changes were attributable to altered food consumption, food intake and energy intake were evaluated. The average daily food intake did not differ among the groups (Figure 1D), suggesting that the interventions did not induce appetite. As expected, daily energy intake was higher in HFD-based groups than in NCD (Figure 1E). However, energy intake did not differ among the HFD, GLU, SBP, and GLU + SBP groups (Figure 1E), indicating that the reduction in body weight was not due to decreased caloric intake within the HFD context. Consistent with the role of adipose expansion in diet-induced obesity, HFD markedly increased Epi-WAT, Per-WAT, and Retro-WAT fat masses relative to NCD (Figure 1F, *p* < 0.05). These increases were substantially attenuated by dietary interventions. Compared with the HFD group, GLU + SBP reduced Epi-WAT, Per-WAT, and Retro-WAT masses from 1.91 ± 0.53, 0.76 ± 0.24, and 1.31 ± 0.52 g to 0.90 ± 0.57, 0.25 ± 0.19, and 0.51 ± 0.33 g, respectively (all *p* < 0.05) (Figure 1F). Accordingly, the fat mass-to-body weight ratio decreased from 10.77 ± 1.16% in the HFD group to 3.34 ± 1.19% in the GLU + SBP group (*p* < 0.05) (Figure 1G), indicating an overall attenuation of HFD-induced adiposity.

HFD feeding also altered circulating lipid indices (Figure 1H–K). Serum TC was elevated in HFD compared with NCD (Figure 1H, *p* < 0.05), whereas GLU, SBP, and GLU + SBP each exhibited significantly lower TC than HFD (Figure 1H, *p* < 0.05). Similarly, serum TG was markedly reduced in the intervention groups compared with the HFD group (Figure 1I). For LDL-C, GLU significantly differed from HFD (Figure 1J), whereas SBP and GLU + SBP did not differ from HFD, as indicated by shared letter annotations.

Collectively, these results indicate that supplementation with oat β-glucan and soybean protein—particularly in combination—attenuates HFD-induced body weight gain and adiposity without changing food intake and improves multiple serum lipid indices under HFD conditions.

### 3.2. Synergistic Improvement of Hepatic Steatosis and Glucose Homeostasis

**Figure 2:**
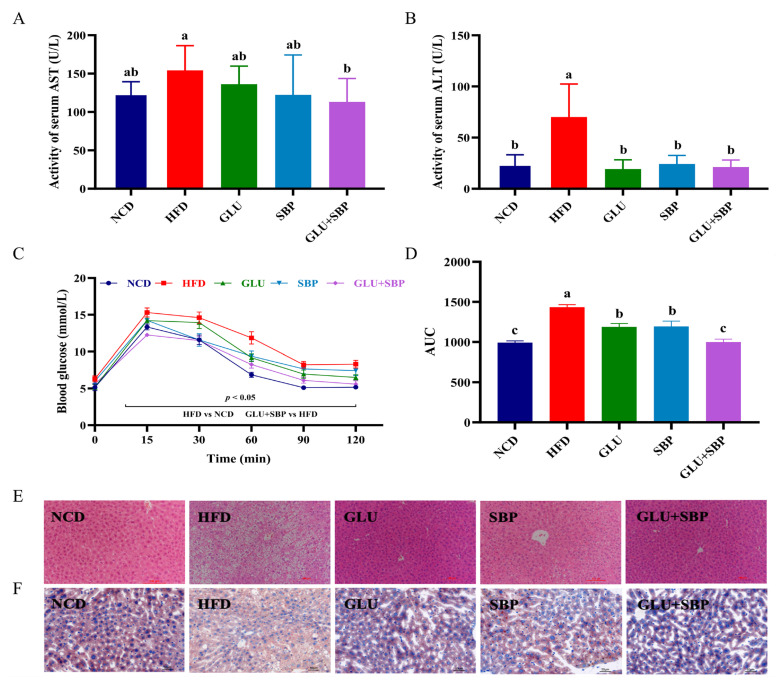
Synergistic protective effects of GLU + SBP combination on liver injury and glucose homeostasis. (**A**) Serum AST activity. (**B**) Serum ALT activity. (**C**) Oral glucose tolerance test curves. (**D**) Area under the curve for OGTT. (**E**) Representative H&E staining images of liver sections (scale bar = 100 μm). (**F**) Representative Oil Red O staining images of liver sections (scale bar = 50 μm). Data are expressed as mean ± SD (*n* = 8). Different letters indicate significant differences (*p* < 0.05) determined via Duncan’s test. NCD: normal control diet, HFD: high-fat diet, GLU: HFD + β-glucan, SBP: HFD + soybean protein, GLU + SBP: HFD + β-glucan and soybean protein combination.

Hepatic injury was evaluated using serum transaminases and liver histology (Figure 2A,B,E,F). Compared with the NCD group, HFD feeding markedly elevated serum AST and ALT activities (Figure 2A,B, both *p* < 0.05), suggesting pronounced hepatocellular injury under obesogenic conditions. Although GLU or SBP alone partially attenuated these increases, the GLU + SBP intervention elicited the greatest improvement in liver function, with transaminase activities shifting closest to the NCD range. Notably, GLU + SBP reduced serum AST activity more effectively than either GLU or SBP alone. AST activity was 113.13 ± 28.50 U/L in the GLU + SBP group, compared with 158.00 ± 30.25 U/L in the HFD group (*p* < 0.05) (Figure 2A), suggesting a combination-specific advantage in attenuating HFD-induced hepatic injury.

Consistent with the biochemical data, representative H&E staining showed marked hepatic steatosis in HFD-fed mice, marked by pronounced vacuolar alterations and disrupted hepatic architecture (Figure 2E, scale bar = 100 μm). These pathological features were visibly alleviated in the intervention groups, particularly in GLU + SBP. Oil Red O staining further confirmed extensive neutral lipid accumulation in the HFD group, whereas lipid droplet deposition was reduced following dietary supplementation (Figure 2F, scale bar = 50 μm), with GLU + SBP exhibiting an apparent decrease in lipid droplet abundance compared with HFD.

Glucose homeostasis was evaluated via the OGTT (Figure 2C,D). HFD-fed mice showed impaired glucose tolerance, as evidenced by elevated glucose levels during the glucose challenge compared with NCD (Figure 2C). Notably, the GLU + SBP intervention markedly improved glucose clearance compared with HFD, as reflected by the OGTT curve (Figure 2C). This improvement was further supported by a significantly lower OGTT AUC in the GLU + SBP group, which decreased from 1434.66 ± 80.56 mmol/L·min in the HFD group to 999.09 ± 95.83 mmol/L·min in the GLU + SBP group (*p* < 0.05) (Figure 2D).

### 3.3. Oat β-Glucan and Soybean Protein Reshape the Gut Microbiota Community Structure and Phylum-Level Features

**Figure 3:**
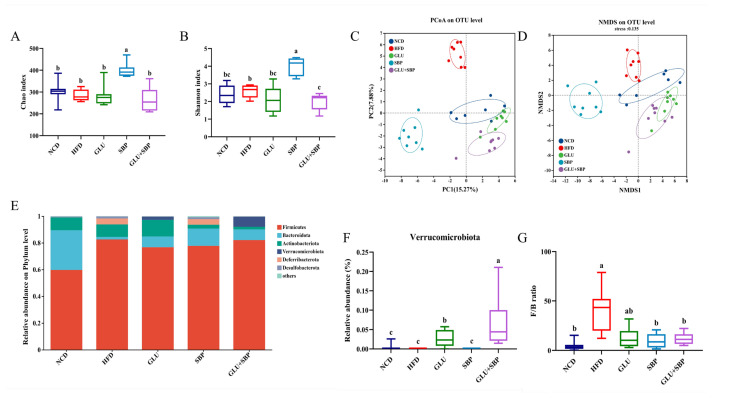
Effects of GLU + SBP combination on gut microbiota diversity and community structure. (**A**) Chao index. (**B**) Shannon index. (**C**) PCoA based on the Bray–Curtis distance. (**D**) NMDS. (**E**) Relative abundance of gut microbiota at the phylum-level. (**F**) Relative abundance of the phylum *Verrucomicrobiota*. (**G**) F/B ratio. Data are expressed as mean ± SD (*n* = 8). Different letters indicate statistically significant differences between the groups (*p* < 0.05) determined by Duncan’s test. NCD: normal control diet, HFD: high-fat diet, GLU: HFD + β-glucan, SBP: HFD + soybean protein, GLU + SBP: HFD + β-glucan and soybean protein combination. Different letters indicate statistically significant differences between the groups (*p* < 0.05) determined by Duncan’s test.

To determine whether dietary interventions modulated the gut microbial ecosystem, fecal microbiota profiles were characterized via 16S rRNA sequencing (Figure 3). Alpha diversity was initially evaluated using richness (Chao1) and diversity (Shannon) indices. In this dataset, HFD did not significantly reduce Chao1 richness compared with NCD, as indicated by shared letter annotations (Figure 3A, *p* < 0.05). Notably, SBP increased Chao1 compared with the other groups (Figure 3A). For Shannon diversity, group differences were also found (Figure 3B): SBP demonstrated the highest diversity, whereas GLU + SBP exhibited a lower Shannon index than the other groups (*p* < 0.05). These results suggest that the interventions affected alpha diversity in a component-dependent manner rather than reflecting a uniform HFD-driven diversity loss.

Beta diversity based on Bray–Curtis dissimilarity further revealed pronounced intervention-associated shifts in community structure. Principal coordinate analysis (PCoA) and NMDS at the OTU level exhibited distinct clustering across the dietary groups (Figure 3C,D; PCoA PC1 = 15.27%, PC2 = 7.8%; NMDS stress = 0.135). The HFD group formed a separate cluster from NCD, thereby supporting a diet-associated community shift. Essentially, GLU, SBP, and GLU + SBP each demonstrated community configurations distinct from HFD, suggesting that supplementation remodeled the gut microbial structure under HFD conditions (Figure 3C,D).

At the phylum-level, the microbiota was dominated by Firmicutes and Bacteroidota across all groups (Figure 3E). Consistent with an obesogenic microbial signature, HFD increased the Firmicutes/Bacteroidota (F/B) ratio compared with NCD (Figure 3G, *p* < 0.05). Such an increase was attenuated by SBP and GLU + SBP, both of which exhibited F/B ratios comparable to that of NCD, whereas GLU exhibited an intermediate pattern (Figure 3G).

Among HFD-based diets, a prominent phylum-level feature was observed for *Verrucomicrobiota*. GLU + SBP markedly increased the relative abundance of *Verrucomicrobiota* compared with HFD and with either single-component intervention (Figure 3F, *p* < 0.05). Similarly, GLU increased *Verrucomicrobiota* compared with groups with low abundance (Figure 3F). Given that *Verrucomicrobiota* includes *Akkermansia*, this enrichment provides a phylum-level basis for subsequent genus level analyses of key taxa associated with metabolic outcomes.

### 3.4. Identification of Key Phylotypes Modulated by β-Glucan, Soybean Protein, and Their Combination

**Figure 4:**
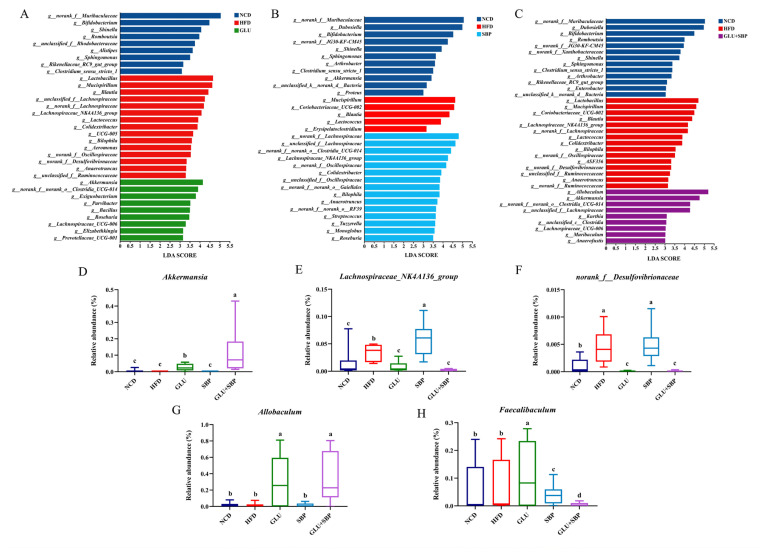
Identification of key phylotypes modulated by specific and synergistic interventions. (**A**–**C**) LEfSe analysis (LDA > 3.0) identifying differentially abundant taxa in the GLU, SBP, and GLU + SBP groups compared with the HFD and NCD group, respectively. (**D**–**H**) Relative abundance of key bacterial genera: (**D**) *Akkermansia* (synergistic responder). (**E**,**F**) *Lachnospiraceae_NK4A136_group* and *norank_f__Desulfovibrionaceae* (GLU-specific responder). (**G**,**H**) *Allobaculum* and *Faecalibaculum* (SBP-specific responder). Data are expressed as mean ± SD (*n* = 8). Different letters indicate statistically significant differences between the groups (*p* < 0.05) determined by Duncan’s test. NCD: normal control diet, HFD: high-fat diet, GLU: HFD + β-glucan, SBP: HFD + soybean protein, GLU + SBP: HFD + β-glucan and soybean protein combination. Different letters indicate statistically significant differences between the groups (*p* < 0.05) determined by Duncan’s test.

To identify the taxa most responsive to each dietary intervention under HFD conditions, LEfSe was applied to compare each intervention group with HFD and NCD (Figure 4A–C). Distinct sets of discriminative features were observed for GLU (Figure 4A), SBP (Figure 4B), and GLU + SBP (Figure 4C) when contrasted with HFD, suggesting that each intervention remodeled the microbial community through a partially nonoverlapping taxonomic signature. To provide a quantitative view of representative responders, we further investigated the relative abundance of selected genera (Figure 4D–H, *p* < 0.05). The combined intervention elicited the most prominent *Akkermansia* enrichment (Figure 4D). *Akkermansia* had the highest abundance in GLU + SBP, exceeding the HFD and single-component groups (*p* < 0.05), suggesting a combination-associated amplification of this key genus.

Taxonomic analysis revealed that the SBP diet specifically enriched the *Lachnospiraceae_NK4A136_group* (Figure 4E), highlighting an SBP-driven expansion of this lineage. Meanwhile, the abundance of *norank_f_Desulfovibrionaceae* (Figure 4F) remained characteristically high under both HFD and SBP treatments. However, it was significantly reduced by GLU and reached its lowest observed levels following the GLU + SBP intervention (*p* < 0.05), underscoring the superior inhibitory efficacy of the combined formulation.

Notably, *Allobaculum* was markedly increased in GLU, with this increase maintained in GLU + SBP (Figure 4G), whereas the levels remained low in NCD, HFD, and SBP (*p* < 0.05). In contrast, *Faecalibaculum* displayed a different pattern (Figure 4H): it was significantly enriched in GLU, whereas its abundance decreased in a stepwise manner in SBP and reached the lowest level in GLU + SBP (*p* < 0.05), suggesting that the combined intervention did not simply mirror all the GLU-driven changes.

Collectively, LEfSe and targeted abundance analyses revealed that β-glucan and SBP modulate the gut microbiota via distinct taxonomic shifts, whereas their combination is characterized by a strong enrichment of *Akkermansia*, maintenance of elevated *Allobaculum*, and marked suppression of *Desulfovibrionaceae*-related taxa.

### 3.5. Association of Gut Microbiota with Host Metabolic Phenotypes and Their Predicted Functional Shifts

**Figure 5:**
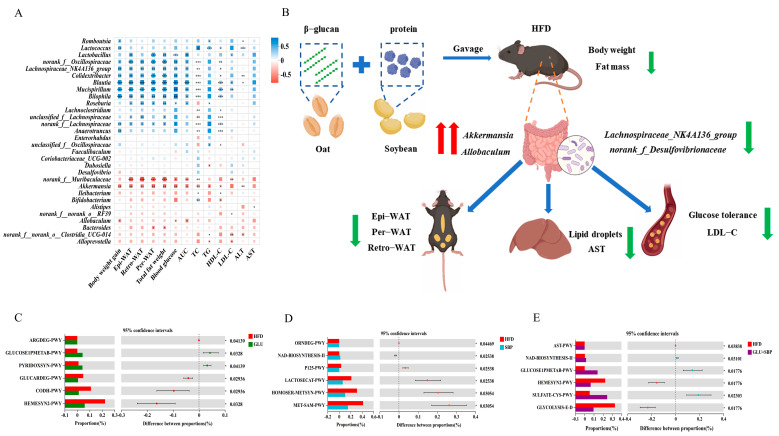
(**A**) Spearman’s correlation heatmap between the key gut bacterial genera and metabolic parameters. (**B**) Integrated mechanism of the GLU + SBP intervention in reshaping gut microbiota to remodel hepatic lipid metabolism and reverse metabolic syndrome. Functional prediction and correlation analysis of gut microbiota. (**C**–**E**) Differentially enriched KEGG pathways between the HFD and intervention groups identified via PICRUSt2 analysis (extended error bar plot). (**C**) HFD vs. GLU, (**D**) HFD vs. SBP, (**E**) HFD vs. GLU + SBP. * *p* < 0.05, ** *p* < 0.01, *** *p* < 0.001. Red indicates a positive correlation, whereas blue indicates a negative correlation.

To link key microbial taxa with host outcomes, Spearman’s correlation analysis was conducted between representative genera and metabolic parameters (Figure 5A). Notably, the taxa highlighted in the community structure analyses (Figure 3) and key phylotype profiling (Figure 4) exhibited coherent association patterns. Specifically, the HFD-associated elevation in the F/B ratio and the low abundance of *Verrucomicrobiota* (Figure 3E–G) were accompanied by reduced levels of the beneficial genus *Akkermansia*, whereas the combined intervention elevated *Verrucomicrobiota* and substantially enriched *Akkermansia* (Figure 3F). Consistently, *Akkermansia* (and the GLU-responsive genus *Allobaculum*, Figure 4G) exhibited negative correlations with obesity- and metabolic syndrome–related traits, including body weight gain, adiposity indices, OGTT AUC, and dyslipidemia-related parameters (Figure 5A, *p* < 0.05). Contrarily, the taxa that tended to co-occur with the HFD-shifted community structure (e.g., *Blautia*, *Mucispirillum*, and *Bilophila*) were positively correlated with these adverse metabolic indices (Figure 5A). Collectively, these association patterns support that the microbiota remodeling captured in Figure 3 and Figure 4, particularly the enrichment of *Verrucomicrobiota*/*Akkermansia* and *Allobaculum* under GLU + SBP, is closely associated with the improvements in glucose homeostasis, lipid profiles, and liver injury markers observed in this study (Figure 1 and Figure 2).

To gain insight into how microbiota remodeling may be associated with the observed metabolic improvements, microbial functions were predicted using PICRUSt2 and compared between the HFD and each intervention group (Figure 5C–E). In the GLU group, several pathways involved in carbohydrate handling and vitamin/cofactor metabolism were enriched compared with the HFD group, including GLUCOSE1PMETAB-PWY and PYRIDOXSYN-PWY (Figure 5C, *p* < 0.05). These predicted shifts are consistent with the improvement of HFD-induced metabolic disturbances observed at the phenotypic level (e.g., reduced adiposity and improved lipid indices; Figure 1 and Figure 2). In the SBP group, pathways associated with redox- and cofactor-associated metabolism were prominently altered, most notably the enrichment of NAD-BIOSYNTHESISII alongside methionine/S-adenosylmethionine-related pathways (HOMOSER-METSYN-PWY, MET-SAM-PWY) (Figure 5D, *p* < 0.05), which is consistent with the reduced liver enzyme activities and improved systemic metabolic status under HFD (Figure 2A,B). Notably, the GLU + SBP combination showed a distinct predicted functional profile, marked by increased NAD-BIOSYNTHESISII and GLUCOSE1PMETAB-PWY, accompanied by reductions in GLYCOLYSIS-E-D and HEMESYN2-PWY (Figure 5E, *p* < 0.05). Collectively, these predicted changes indicate that the combined intervention may simultaneously modulate microbial carbohydrate-related functions and cofactor/redox-associated metabolic capacity, thereby providing a functional context for the superior enhancements in glucose tolerance (lower OGTT AUC) and hepatic injury/steatosis under HFD (Figure 2C–F).

As summarized in Figure 5B, GLU + SBP elicited the most consistent metabolic improvement under HFD. Compared with HFD, GLU + SBP robustly attenuated body weight gain and white adipose burden, accompanied by a substantial decrease in hepatic lipid deposition. These morphological benefits were paralleled by enhanced liver function, with transaminase activities shifting toward the NCD range, and by improved glucose homeostasis, reflected by enhanced glucose tolerance. Collectively, the integrated phenotype in Figure 5B highlights that the combined intervention simultaneously alleviated obesity, ectopic lipid accumulation, hepatic injury, and insulin resistance, thereby supporting a combination-specific advantage of GLU + SBP over single-component supplementation.

## 4. Discussion

The traditional dietary combination of cereals with legumes is widely recognized for its health benefits; however, whether specific bioactive macromolecules confer synergistic advantages remains unclear. This study demonstrates that the combined GLU + SBP intervention provides comprehensive protection against HFD-induced metabolic dysregulation in C57BL/6J mice. Compared with the HFD group, GLU + SBP significantly attenuated body weight gain and adiposity without influencing caloric intake, improved multiple serum lipid parameters, and alleviated hepatic steatosis, as evidenced by enhanced liver histomorphology and reduced lipid deposition. Notably, GLU + SBP elicited combination-specific enhancements in key metabolic endpoints, with greater reductions in serum AST activity and lower OGTT AUC values than either GLU or SBP alone (*p* < 0.05), thereby exerting synergistic effects exceeded those of the individual components. Concurrently, 16S rRNA sequencing revealed distinct gut microbial community remodeling under GLU + SBP, characterized by normalization of the F/B ratio toward levels observed in standard chow-fed mice, substantial enrichment of *Verrucomicrobiota* (particularly *Akkermansia*), sustained elevation of *Allobaculum*, and suppression of *Desulfovibrionaceae*-associated taxa. Functional inference using PICRUSt2 further identified intervention-specific modulation of microbial pathways involve in carbohydrate metabolism and redox/cofactor processes. These integrated phenotypic and microbial findings support the concept that combined soluble fiber and plant protein formulations may confer coordinated metabolic benefits under obesogenic conditions, thereby providing a preliminary basis for developing cereal–legume-inspired functional food strategies.

Under HFD conditions, the GLU + SBP combination produced the most consistent attenuation of body weight gain and reduction in adiposity indices despite comparable caloric intake among groups, indicating that these effects are attributable to metabolic partitioning rather than caloric restriction. This phenotypic response aligns with emerging animal studies showing that oat β-glucan mitigates diet-induced obesity and adipose tissue expansion via host–microbiota–tissue crosstalk mechanisms (e.g., reduced ectopic lipid deposition and enhanced metabolic homeostasis), independent of caloric intake modulation [32]. Similarly, soy protein–dominant diets have been shown to modulate metabolic outcomes in obesity-related contexts, with some studies reporting enhanced insulin sensitivity despite minimal weight loss, suggesting that dietary protein sources may uncouple adiposity from metabolic risk [33]. Collectively, this evidence supports the interpretation that the GLU + SBP combination may redirect nutrient metabolism toward enhanced metabolic efficiency—characterized by reduced fat storage and lower adipose burden—rather than simply limiting caloric intake.

GLU + SBP improved multiple serum lipid indices while concurrently reducing hepatic lipid deposition, suggesting coordinated improvement in lipid trafficking and ectopic fat accumulation. Previous systematic reviews and clinical studies have consistently associated oat consumption, particularly β-glucan-rich formulations, with favorable changes in dyslipidemia-related profiles, including reductions in circulating TC and LDL-C [34]. This is further supported by a recent randomized controlled trial in individuals with metabolic syndrome, showing that oat-based dietary intervention reduced serum TC and LDL-C and was associated with gut microbiota-derived phenolic metabolites [35]. Mechanistically, animal studies suggest that β-glucan can modulate the gut–liver axis, including bile acid-related pathways, providing a plausible biological basis for reduced hepatic lipid loading under GLU-containing diets [15]. Recent MAFLD-focused reviews further emphasize the roles of gut microbial metabolites, intestinal barrier integrity, bile acid signaling, and immune–metabolic crosstalk in hepatic lipid regulation [36]. On the legume side, dietary soy protein has been shown to attenuate liver steatosis in obese models, accompanied by microbiota alterations [18]. In addition, recent evidence highlights a bidirectional relationship between soy foods and the human gut microbiome, whereby soy-derived compounds may be transformed into bioactive metabolites while also shaping microbial composition and activity [37]. Collectively, our findings are consistent with both preclinical and human evidence, suggesting that enhanced regulation of lipid metabolism coupled with reduced ectopic lipid deposition, through which dietary fiber and plant proteins synergistically interact to mitigate hepatic abnormalities.

Beyond morphological changes, GLU + SBP administration significantly modulated transaminase levels toward the reference range associated with MAFLD-related disease, particularly evident in the improved markers of hepatocellular injury. This observation supports the established paradigm whereby gut-derived inflammatory mediators and bile acid–sensing pathways influence hepatic injury progression in dietary steatohepatitis. Consistent with this mechanism, a preclinical investigation demonstrated that dietary oat β-glucan attenuates the progression of steatotic liver disease through microbiota-dependent signaling pathways [15], thereby strengthening the hypothesis that fiber-mediated gut-liver axis communication may mitigate hepatic stress responses. Notably, *Desulfovibrionaceae*/*Desulfovibrio* lineages have been mechanistically linked to exacerbated hepatic steatosis phenotypes: experimental evidence revealed that *Desulfovibrio piger* exacerbates steatofibrotic lesions in HFD-fed mouse models while exhibiting increased relative abundance in fecal microbiota of obese pediatric MAFLD patients—findings that substantiate clinical interpretations of its suppression as potentially beneficial [25]. Contemporary systematic reviews further consolidate emerging evidence connecting *Desulfovibrio* expansion to both hepatic lipid accumulation and systemic inflammatory activation, albeit with recognized contextual dependencies [38]. Together, these convergent mechanisms support the interpretation that the observed biochemical improvements (normalization of ALT/AST) are physiologically correlated with coordinated reductions in hepatic lipid content and attenuated pro-inflammatory gut ecosystem dynamics [2,15,25,38].

The GLU + SBP intervention demonstrated superior efficacy in reducing OGTT AUC compared to its individual components, suggesting a synergistic enhancement of glucose metabolism under HFD conditions. Recent mechanistic studies have demonstrated that dietary oat β-glucan attenuates HFD-induced insulin resistance and intestinal dysfunction, highlighting its capacity to modulate glucose homeostasis through gut-mediated mechanisms independent of weight loss [16]. Moreover, emerging evidence from fermentation studies supports the concept of non-additive metabolic signaling in fiber-protein co-administration; these studies reveal that protein co-ingested with specific dietary fibers enhances butyrate production while reshaping microbial metabolite profiles, thereby providing mechanistic insights into substrate-dependent regulation of fermentation outputs relevant to glycemic control [39]. These mechanistic observations align with our experimental findings, which indicate augmented glucose tolerance beyond individual ingredient effects, potentially mediated by coordinated modifications in nutrient absorption dynamics, gut-derived metabolic signaling, and subsequent hepatic and peripheral insulin sensitivity.

A prominent microbial feature of GLU + SBP was the marked enrichment of *Verrucomicrobiota*/*Akkermansia* alongside suppression of *Desulfovibrionaceae*-related taxa. From a translational perspective, *Akkermansia muciniphila* has emerged as a next-generation probiotic candidate associated with metabolic resilience. Notably, a randomized trial in obese patients with T2D demonstrated that supplementation with *A. muciniphila* at a dose of 1 × 10^10^ bacteria/day for 3 months produced metabolic benefits that may depend on baseline intestinal abundance levels, highlighting its potential in precision nutrition strategies and offering mechanistic insights into observed clinical responses [40]. Mechanistic studies have demonstrated that *Akkermansia* is capable of modulating mucus layer integrity and gut barrier function, thereby mitigating metabolic endotoxemia and systemic inflammation—pathophysiological processes intrinsically associated with hepatic injury and insulin resistance [41]. In this context, the *Akkermansia* enrichment specifically observed in GLU + SBP represents a plausible ecological signature associated with the most pronounced improvements in OGTT AUC and transaminase levels, although direct causal relationships require experimental validation.

### 4.1. Strengths

This study also has several strengths. First, the experimental design directly compared GLU, SBP, and their combination within an isocaloric HFD framework, allowing the combined intervention to be interpreted against both in-dividual components rather than against HFD alone. Second, food intake and energy intake were monitored, supporting the conclusion that the observed metabolic improvements were not simply attributable to reduced caloric consumption. Third, the study integrated phenotypic, biochemical, histological, and 16S rRNA sequencing readouts, providing a multi-level preclinical dataset linking host metabolic outcomes with microbial community shifts. Finally, the use of readily available dietary macromolecules—oat β-glucan and soy protein isolate—enhances the practical relevance of the findings for future functional food development.

### 4.2. Limitations

This study has several limitations that should be acknowledged. First, the use of a mouse model restricts direct extrapolation to human physiology because of interspecies differences in nutrient metabolism, immune responses, and gut microbiota composition. Second, the intervention was evaluated at a single dose level, precluding assessment of dose–response relationships, thresholds, and potential saturation effects for GLU, SBP, and their combination. Third, although 16S rRNA sequencing revealed marked alterations in gut microbial structure, functional microbial outputs such as short-chain fatty acids, bile acids, indole derivatives, and other microbial metabolites were not quantified. Fourth, the study primarily reports phenotypic, biochemical, histological, and taxonomic endpoints, whereas key molecular pathways related to lipid metabolism, inflammation, insulin sensitivity, intestinal barrier integrity, and hepatic glucose/lipid signaling were not directly validated. Fifth, the sample size may limit statistical power for some endpoints, and additional details on blinding during outcome assessment should be provided where applicable. Finally, long-term durability, safety, feasibility, and translational efficacy in human populations were not assessed.

### 4.3. Future Perspectives

Future studies should focus on elucidating the molecular mechanisms underlying the complementary effects of β-glucan and soy protein, particularly by investigating key metabolic and inflammatory signaling pathways such as AMPK, SIRT1, NF-κB, insulin signaling, bile acid receptors, and hepatic lipid oxidation pathways. Integrative multiomics approaches combining shotgun metagenomics, metabolomics, transcriptomics, and targeted biochemical assays are warranted to better characterize microbial functional capacity and host metabolic responses. Causal validation should include fecal microbiota transplantation, antibiotic depletion or gnotobiotic models, targeted *Akkermansia* supplementation/depletion, and direct measurement of SCFAs, bile acids, intestinal barrier markers, and inflammatory cytokines. Longitudinal animal studies are also needed to determine the durability of the metabolic improvements and their effects on disease progression. Importantly, well-designed human intervention trials should evaluate dose–response relationships, safety, acceptability, and feasibility of translating the GLU + SBP combination into functional food formulations for cardiometabolic disease prevention and management.

## 5. Conclusions

In summary, the GLU + SBP co-formulation exhibits synergistic efficacy in mitigating HFD-induced metabolic dysregulation, significantly outperforming individual interventions. A key innovation of this rational design of dietary fiber–plant protein formulations lies in its ability to achieve systemic metabolic improvements solely through enhanced diet quality, independent of caloric restriction. These beneficial effects were accompanied by marked remodeling of the gut microbiota, characterized by enrichment of *Akkermansia* and reduction in *Desulfovibrionaceae*-related taxa, suggesting that gut microbial modulation may contribute to the metabolic improvements observed in HFD-fed mice. Collectively, this study provides preclinical evidence supporting the rational design of dietary fiber–plant protein formulations for obesity-related metabolic disorders.

## Figures and Tables

**Table 1:** Composition of the experimental diets.

Component (g/kg)	NCD	HFD	GLU	SBP	GLU + SBP
β-Glucan	0	0	64.45	0	64.45
Soybean protein	0	0	0	212.67	212.67
Casein	189.56	258.45	256.28	73	70.83
L-Cystine	2.84	3.88	3.88	3.88	3.88
Corn starch	479.79	0	0	0	0
Maltodextrin 10	118.48	161.53	154.5	161.53	154.5
Sucrose	73.74	88.91	88.91	88.91	88.91
Cellulose, BW200	47.39	64.61	12.02	64.22	11.63
Soybean oil	23.7	32.31	32.18	27.42	27.29
Lard	18.96	316.6	316.6	316.6	316.6
Mineral mix S10026	9.48	12.92	12.92	12.92	12.92
Calcium hydrogen phosphate	12.32	16.8	16.8	16.8	16.8
Calcium carbonate	5.21	7.11	7.11	7.11	7.11
Potassium citrate, 1 H_2_O	15.64	21.32	21.32	21.32	21.32
Vitamin mix, V10001	0.95	12.92	12.92	12.92	12.92
Choline tartrate	1.9	2.58	2.58	2.58	2.58
FD&C blue dye	0.01	0	0	0	0
FD&C yellow dye	0.04	0	0	0	0
Total (g)	1000	1000	1002.47	1021.88	1024.41
Total kcal	3845.31	5242.88	5242.83	5242.89	5242.84

NCD: normal control diet, HFD: high-fat diet, GLU: HFD + β-glucan, SBP: HFD + soybean protein, GLU + SBP: HFD + β-glucan and soybean protein combination.

## Data Availability

The original contributions presented in the study are included in the article/Supplementary Materials, further inquiries can be directed to the corresponding author.

## Supplementary Materials

The following supporting information can be downloaded at: https://www.mdpi.com/article/10.3390/nu18101571/s1, Table S1: The basic composition of β-glucan and soy bean protein (dry weight basis).

## Abbreviations

The following abbreviations are used in this manuscript:

HFD	high-fat diet
OGTT	oral glucose tolerance test
AUC	area under the curve
MAFLD	metabolic and fatty liver disease
TC	total cholesterol
TG	triglycerides
HDL-C	high-density lipoprotein cholesterol
LDL-C	low-density lipoprotein cholesterol
AST	aspartate aminotransferase
ALT	alanine aminotransferase
NCD	Normal control diet
GLU	HFD supplemented with oat β-glucan
SBP	HFD supplemented with soybean protein isolate
GLU + SBP	HFD supplemented with both oat β-glucan and soybean protein isolate
Epi-WAT	epididymal
Per-WAT	perirenal
Retro-WAT	retroperitoneal
OTUs	operational taxonomic units
PERMANOVA	permutational multivariate analysis of variance
LEfSe	Linear discriminant analysis effect size
LDA	linear discriminant analysis
KEGG	The Kyoto Encyclopedia of Genes and Genomes
ANOVA	analysis of variance
PCoA	Principal coordinate analysis
