# Supplementary material for: Combined Oat β-Glucan and Soy Protein Isolate Reprogram Gut Microbiota and Improve Metabolic Dysfunction in Diet-Induced Obesity
Source: Nutrients. 2026 May 15;18(10):1571. doi: 10.3390/nu18101571 (PMC13209952; doi:10.3390/nu18101571)
Supplement: Supplementary file 1 [file nutrients-18-01571-s001.zip › nutrients-4286170-supplementary.pdf]

Table S1 The basic composition of β-glucan and soy bean protein (dry weight basis)

| | Protein (g/100g) | Fat (g/100g) | Carbohydrate (g/100g) | Crude fiber (g/100g) | Moisture (g/100g) |
|---|---|---|---|---|---|
| β-glucan | 3.36 ± 0.03 | 0.20 ± 0.00 | 10.09 ± 0.00 | 81.50 ± 0.55 | 3.84 ± 0.03 |
| soy bean protein | 87.20 ± 0.10 | 2.30 ± 0.10 | 0.00 ± 0.00 | 0.18 ± 0.01 | 5.21 ± 0.03 |
